# Study on hormone induction of the male parent and embryonic development, gonad differentiation, and growth of “All-female No.1” *Culteralburnus*

**DOI:** 10.1016/j.heliyon.2024.e33414

**Published:** 2024-06-21

**Authors:** Shun Cheng, Mei-li Chi, Shi-li Liu, Jian-bo Zheng, Wen-ping Jiang, Xiao-ying Hang, Miao Peng, Fei Li

**Affiliations:** Zhejiang Institute of Freshwater Fisheries, Agriculture Ministry Key Laboratory of Healthy Freshwater Aquaculture, Key Laboratory of Freshwater Aquatic Animal Genetic and Breeding of Zhejiang Province, Huzhou, Zhejiang, 313001, China

**Keywords:** Topmouth culter, Pseudo-male, All-female, Ovary, Growth

## Abstract

Female *Culter alburnus* was faster in growth rate than males. In this study, the gynogenetic G2 and the pseudo-male G2' were used as the female and male parents, respectively, to breed a new national variety “All-female No.1” *C. alburnus* (AFC). Hormone induction, embryonic development, gonadal differentiation, and growth of AFC were studied. The results showed induction with low concentrations of 17α-methyltestosterone in a indoor-net cage culture was not effective. Under the stimulation of 17α-methyltestosterone, some gonads had a tendency to transform into testis, but not completely. There were three types of gonads in 5-month-old and four types of gonads in 12-month-old fishes, however, they all differentiated into ovaries in 15-month-old fishes. Testosterone propionate and high concentrations of 17α-methyltestosterone in pond culture induction had a good effect resulting in ①a functional pseudo-male with normal testis development that could successfully extrude semen during the breeding period, ②a pseudo-male with normal testis development, but could not extrude semen, and ③the appearance of intersexual glands. The second experiment revealed that with common fish, all-female fish embryo had normal embryonic development. The development time and morphological characteristics of each stage were similar, but the development of the all-female embryo was slightly slower than the common embryos. The gonad differentiation of the all-female embryo were normal and none differentiated into testis, which indicated that all-female could ensure the female sex without affecting the normal gonad differentiation. The correlation between body weight, length, and month-age of all-female and common fish was strong. The all-female had faster growth rate and more uniform growth specification than the common fish. Therefore, the use of testosterone propionate and high concentrations of 17α-methyltestosterone in pond culture induction could avoid complete degeneration of gonads into ovaries. The all-female embryo had the advantages of normal embryonic development and gonadal differentiation, faster growth, and uniform growth specification.

## 1 Introduction

*Culter alburnus* is an economically valuable freshwater fish in China [1,2]. The growth rate of females is faster than that of males [2,3], thus, breeding all-female fish can improve yield. In view of this, our unit had cultivated a new national variety “All-female No.1” *C. alburnu*s (AFC) using population breeding individuals as parents, through gynogenesis combined with pseudo-male induction [3].

The effect of hormone induction could be analyzed through histological and gonadal differentiation studies. Liu et al. [4] compared the effect of different 17α-methyltestosterone (17α-MT) concentrations on the gonad of *Pseudorasbora parva* and reported that 50 ng/L 17α-MT had the most significant effect on gonad index and histology, whereas a concentration of 100 ng/L 17α-MT would inhibit the development of gonads and hinder the maturation of germ cells. Pawlowski et al. [5] and Kang et al. [6] had also reached similar conclusions in their studies on *Pimephales promelas* and *Oryzias latipes*. Lin et al. [7] studied the immersion treatment of gynogenetic *Nibea albiflora* using 0.5 μg/L 17α-MT and reported that the gonad of 55-day-old fish showed oocyte and spermatic duct primordia at the same time, indicating that exogenous 17α-MT inhibited oocytes and promoted the development of male germ cells. Li et al. [8] found that *Epinephelus akaara* treated with 17α-MT underwent sex reversal wherein the spermatogonia and spermatocytes became the dominant cells in the gonad, and eventually produced semen. They also found that the male semen produced from the sex reversal induced by high-dose or long-term 17α-MT treatment had no insemination ability. However, at present, studies on the effects of androgens on fish gonads mainly focused on 17α-MT, and generally immersion treatment of fry had limited practical effect, which was not conducive to large-scale production.

In order to compared the effects of different hormone treatments, we should study the histological differentiation trend of gonads. Nakamura et al. [9] and Strussmann and Nakamura [10] believed that a large number of germ cells in the primordial gonad of teleost fishes would develop into ovaries, and the ovaries developed earlier than the testes. In the process of gonad differentiation of *Clarias fuscus*, the gonad volume that would develop into ovaries in the future rapidly increases, however, the posterior end of the gonad that would develop into the testis was slender and long [11]. Chen et al. [12] found that if the gonad of the *Misgurnus anguillicauatus* was flat, its back was close to the peritoneal epithelium and close to one side or the middle, it would be differentiated into ovary. When the gonad was pear-shaped, the dorsal side became narrow and stalk-shaped, hanging from the mesentery to the peritoneal epithelium. As it was located on both sides of the body, it would eventually differentiate into the testis. Among carp, *Carassius auratus* gonads which were characterized by having a primary cavity, relatively large volume, and having the back close to the peritoneal epithelium would differentiate into ovaries. The gonads that were characterized as having no primary cavity, small volume, pear-shaped, and were vertically suspended through the mesentery would differentiate into testis [13]. The gonads of *Vimba vimba* which were spindle-shaped and located close to the abdominal wall would differentiate into ovaries. However, the gonads that were pear-shaped and hanged from the mesentery to the peritoneal epithelium would differentiate into testis [14].

As a newly cultivated cultivar, the basic biological data of AFC was weak, so a series of studies were urgently needed to grasp its biological characteristics, which could provide reference for breeding. Among them, embryo development, growth (growth rate, specification uniformity), gonad differentiation, and several other traits were important indicators for evaluating species characteristics. In terms of embryo development research, there had been some related research reports on *C. alburnus*. Zhang et al. [15] made a preliminary observation on the embryo development of wild *C. alburnus* in Xingkai Lake, the result showed that at 22–24 °C, the fertilized eggs made the first cell division after an hour 20 min, after 12 h organs started to develop, after 28 h larvae got out of egg membrane. Gu et al. [16] systematically observed the embryonic and post-embryonic development of *C. alburnus* in Taihu Lake. The results showed that at water temperature of 23–25 °C, the process of embryonic development could be divided into 19 stages, which was artificially cultivated under pool culture conditions. Shao et al. [17] systematically observed the embryonic and larval development of *C. alburnus* in Miaohu area, the results are basically consistent with previous research findings. Huang et al. [18] studied body weight and body length changes of *C. alburnus* in the Songhua River. Huang et al. [19] studied the relationship between body length and body weight and the related growth model of *C. alburnus* in Shanmei Reservoir. These two studies both reflected well the growth process and its changing patterns of *C. alburnus.* Histologic observation of fish gonads using was important for studying the characteristics of fish reproduction and development, and could provide background information for reproductive biology and aquaculture technology [10]. Among culter subfamily fishes, Cao et al. [20] studied the annual change process of ovarian development of *Ancherythroculter nigrocaud* using tissue sections and measurement statistics of important reproductive biological parameters, providing basic data for optimizing artificial propagation technology.

The acquisition of AFC required that gynogenetic fish and pseudo-males be used as parents for mating reproduction, thus, it was of great significance to study the effects of different hormone treatments on pseudo-male induction. To compare the effects of different hormone treatments, we studied the histological differentiation trend of gonads after hormone induction.

We used two hormone feeding methods to induce gynogenetic *Culter alburnus* fry and studied the differentiation trend of gonads after hormone treatment from the histological perspective. Additionally, we discussed and compared the effects of different hormone induction methods to guide and improve pseudo-male induction technology, optimized the all-female fish cultivation technology, and provided a theoretical basis for future molecular genetic related research. In short, this study discusses and analyzes the characteristics of embryo development, growth rate, coefficient of variation, gonad differentiation, sex, and other aspects of the all-female *C. alburnus* to provide assistance for the cultivation of a new breed of *C. alburnus* that had the advantages of being all-female, rapid and uniform growth, good genetic consistency, and suitable for large-scale production.

## 2 Materials and methods

### 2.1 Experiment I

The experiments were conducted at the Zhejiang Institute of Freshwater Fisheries, Huzhou, China. *C. alburnus* were obtained from females at the Zhejiang Institute of Freshwater Fisheries, Huzhou, China. *C. alburnus* fry (*C. alburnus* in the Zhejiang Institute of Freshwater Fisheries through population breeding as female parents, and stimulate by inactivating carp sperm) produced by gynogenesiswere placed in a pond with an area of 3 m^2^ or an outer pond, and two hormone induction methods were used on gynogenetic fish, respectively. Gynogenesis in this study is through the induction of gynogenesis fry by cold shock (4 min after insemination, 2 °C ice water cold shock treatment for 15 min) inhibiting the excretion of the second polar body with genetically inactivated carp sperm as the activation source [3,21].

Low concentration 17α-MT was used for indoor-net cage culture induction. The experimental fish was newly hatched fry produced through gynogenesis technology. Feeding mixed bait was placed in the cement pool of the indoor, with feeding concentrations of 10, 25, 40, and 60 μg/g 17α-MT, respectively. We selected these four concentrations based on our team's previous research results [1]. Each hormone concentration was set with a comparative test of 40 days of hormone feeding +70 days of normal feeding (no hormone) (10-1, 25-1, 40-1, 60-1), 60 days of hormone feeding + 50 days of normal feeding (10-2, 25-2, 40-2, 60-2), and 80 days of hormone feeding + 30 days of normal feeding (10-3, 25-3, 40-3, 60-3). Treatments with different concentrations and treatment times were divided across 12 experimental groups. There were 3 replicates in each group. After 3 months of cultivation in the indoor, the fish were transferred to the cage for further cultivation. Tissue sections of the gonads of *C. alburnus* at 2, 3, 5, 12, and 15 months of age were prepared, Tissue sections of the gonads were stored in Bouin's solution, embedded in conventional paraffin, continuously sliced, with a slice thickness of 6 μm. HE staining, microscopic observation and photography, and the gonad development and male ratio were observed and determined. 20 fish per experimental group for each sampling.

Testosterone propionate and a high concentration (30 → 140 μg/g) 17α-MT was used for pond culture induction to induce pseudo-males. Namely, 1 g/L testosterone propionate was sprayed in the pond every day for 15 days. The hormone concentration is selected based on the previous research results of our team [1]. Then the larvae were fed 17α-MT once they could take feed. The hormone concentration used in feeding increased gradually with the growth of fish (30 μg/g→60 μg/g→100 μg/g→120 μg/g→140 μg/g). The induction time of 17α-MT was 105 days. In November, the fish were fed a diet without hormone. Samples were taken at 5, 12, 24, and 36 months of age. 20 fish per experimental group for each sampling. Tissue sections of the gonads were prepared and the gonad development and male ratio were observed and determined.

### 2.2 Experiment II

#### 2.2.1 Embryonic development

The sperm of the pseudo-male G2' and the common-male were inseminated with freshly extruded G2 and common-female eggs using the dry method, respectively. Briefly, the semen was added into the egg holding culture dish, stirred evenly, and a proper amount of pond water was added. After approximately 1 min of shaking, all-female and common fish group were established, which were used as three parallel experiments. All embryos were incubated in purified water at 24–26 °C. The water was changed every 2 h. Samples were taken regularly according to each stage of embryo development. 30 live fish were measured at each stage. The morphological characteristics of each stage of embryo development were observed under the microscope (Table 1). The point at which half of embryos developed to a certain stage was taken as the embryonic development time of this stage.

**Table 1:** Embryonic development of *C. alburnus*.

Developmental stage	Water temperature/°C	Time		Characteristics
		AFC	Common fish	
Fertilized eggs	24	00:00	00:00	Spherical, greenish-grey, and slightly sticky
Blastoderm stage	24	12 min	10 min	The fertilized egg absorbs water and swells, and the protoplasm gradually concentrates to the animal pole, forming a blastoderm and gradually bulges
Two cells stage	24	25 min	22 min	A cleavage groove is formed at the top center of the blastoderm, through which two cells are produced
Four cells stage	24	33 min	30 min	The cleavage groove and the first vertical meridian split into four cells
Eight cells stage	24.5	40 min	37 min	The division direction is parallel to the first cleavage, and the original four cells divide into eight cells symmetrically
Morula stage	24.5	2 h 10 min	2 h	The cells continue to divide, the mitotic sphere becomes smaller and smaller, and the cell mass bulges in a mulberry shape
Early blastocyst	25	2 h 55 min	2 h 30 min	The boundaries of cells between blastomeres are gradually blurred. The blastodisc is located on the yolk. A relatively flat area, namely the yolk syncytial layer, can be observed
Middle blastula	25	3 h 30 min	3 h 15 min	The cell boundary disappears, the number of cells increases gradually, and the blastoderm becomes shorter
Late blastula	26	4 h 20 min	4 h 10 min	The cells on the surface of the blastocyst surround the yolk, the blastodisc becomes arcuate, and the yolk syncytial layer becomes bell-shaped or dome-shaped, indicating that gastrulation is about to begin
Early gastrulation	26	5 h 25 min	5 h 05 min	Hypodermis about 1/2, with embryo ring and back lip
Middle gastrulation	26	6 h 15 min	5 h 55 min	Hypodermis about 2/3, embryo shield appears
Late gastrulation	26	7 h	6 h 35 min	Hypodermis about 3/4
Neuro-embryonic stage	26	7 h 35 min	7 h 15 min	The embryonic nerve plate is formed, leaving a small yolk plug, and the front end of the embryo body is swollen with brain bubbles
Blastopore obturation	26	8 h 35 min	8 h 15 min	The embryo layer completely encloses the yolk sac, and the blastopore is closed
Sarcomere stage	26	9 h 35 min	9 h 20 min	2–3 pairs of fuzzy somites appear in the middle of embryo
Ocular basal stage	26	10 h 35 min	10 h 25 min	The head of the embryo body is slightly raised, and the oculogenin appears
Saccular stage	26	11 h 35 min	11 h 25 min	Oval eye sacs appear on both sides of embryo head
Olfactory plate stage	26	12 h 05 min	11 h 55 min	Concave lines appear in the middle of the eye bag, and fuzzy circles appear below the eyes
Tail-bud stage	25.5	12 h 25 min	12 h 15 min	Tail buds appear on the ventral surface of the rear part of the embryo body, which is in the shape of a round cone
Auditory capsular stage	25.5	12 h 55 min	12 h 45 min	“Bubble” ear sacs appear on both sides of the hindbrain
Occurrence of caudal fin stage	25.5	14 h 05 min	13 h 55 min	The tail of the embryo leaves the yolk sac and the tail fin appears
Crystal appearance stage	25	15 h 15 min	15 h 05 min	Round crystals can be seen in the eye bag
Cardiac stage	25	16 h	15 h 35 min	Cardiac primordium appears at the rear ventral surface
Otolith stage	25	18 h 05 min	17 h 45 min	Shiny round granules appear in the ear sac
Heartbeat stage	25	23 h 15 min	22 h 55 min	The heart starts to beat
Earlier exfoliation stage	25	25 h 10 min	24 h 40 min	The embryo rotates in the egg membrane, and the head and tail constantly collide with the egg membrane
Hatching stage	25	26 h 30 min	25 h 25 min	Hatching begins

#### 2.2.2 Observation and verification of gonad differentiation

The gonads were taken from 2, 4, 6, and 13-month-old all-female and common *C. alburnus*. After fixation with Bouin's solution, routine paraffin embedding was conducted and the sections were continuously cross cut with a thickness of 6 μm. After HE staining, the sections were sealed, observed, photographed under the microscope, and the formation of the vas deferens or ovarian cavity was taken as the anatomical mark. The appearance of oogonia (or spermatogonium) and oocyte (or spermatocyte) as cytological markers was used to determine the gonadal development and gender.

#### 2.2.3 Growth assay

All-female and common *C. alburnus* were bred under the same environmental conditions. Samples were taken at the ages of 2, 6, 13, and 17 months, and 30 fish were measured in each experimental group (Table 2). The body weight and length were measured The following values were computed: coefficient of variation = SD/MN × 100 %; relationship between body length and body weight: W = aL^b^; the growth curve of body weight and month W = at^b^; and the growth curve of body length and month: L = at^b^ where W is the body weight (g), L is the body length (cm), SD is the standard deviation, MN is the average value, t is the month age, and a and b are constants [22,23].

**Table 2:** The growth indexes between all-female and common *C. alburnus*.

Month-old	Group	Body weight/g	Body length/cm	Variation of body weight/%	Variation of body length/%
2	All-female fish	0.48 ± 0.25^a^	3.41 ± 0.61^a^	52.24	17.89
	Common fish	0.47 ± 0.20^a^	3.48 ± 0.49^a^	43.10	14.16
6	All-female fish	4.16 ± 1.94^b^	6.80 ± 1.07^b^	46.60	15.77
	Common fish	4.10 ± 1.92^b^	6.79 ± 1.24^b^	46.79	18.29
13	All-female fish	105.19 ± 12.71^c^	21.27 ± 0.82^c^	12.08	3.87
	Common fish	103.51 ± 17.95^c^	20.71 ± 1.16^c^	17.35	5.61
17	All-female fish	254.66 ± 88.27^d^	28.71 ± 2.53^d^	34.66	8.81
	Common fish	234.55 ± 140.93^e^	26.01 ± 5.90^e^	60.08	22.69

All data are presented as the mean ± SD. The different superscript letters indicate significant differences (*P* < 0.05).

### 2.3 Statistical analysis

All data were presented as mean ± SD. After, arc-sine transformation, the obtained data for each experiment underwent analysis of variance (ANOVA) using SPSS software version 17.0 (IBM, Armonk, NY, USA) to determine group differences. Tukey's multiple comparison *post-hoc* test was performed when significant differences were detected. Statistical significance was set at *P* ≤ 0.05 for all analyses.

## 3 Results

### 3.1 Experiment I

#### 3.1.1 Effect of low concentration 17α-MT on indoor-net cage culture induction

**Fig. 1:**
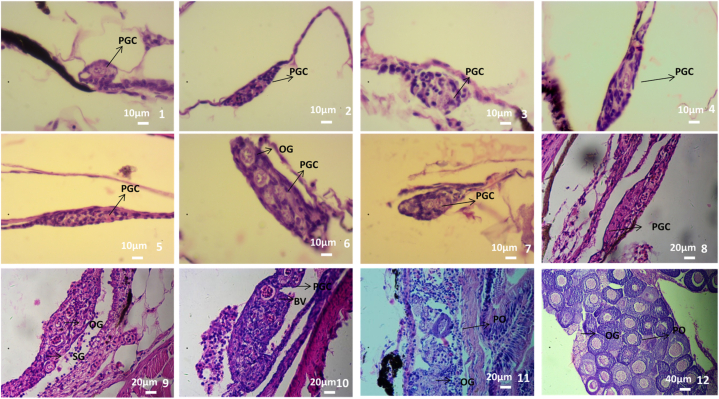
Gonad differentiation after treatment with low concentration 17α-MT in indoor-net cage culture induction of *C. alburnus* (The direction of the cut section is consistent). 1. The gonad of 2-month-old fish (60 concentration group), pear-shaped, with one end connected to the mesentery, which was suspended in the mesentery. 2. The gonad of 2-month-old fish (40 concentration group), spindle-shaped, both ends connected with mesentery. 3. The gonad of 3-month-old fish (40 concentration group), pear-shaped. 4. The gonad of 3-month-old fish (40 concentration group), spindle-shaped. 5. The gonad of 5-month-old fish (40 concentration group), the gonad was elongated on both sides, shoulder pole-shaped. 6. The gonad of 5-month-old fish (40 concentration group), the volume continued to increase, widening at both ends, forming a cylindrical shape, with multiple gonocytes and oogonia. 7. The gonad of 5-month-old fish (25 concentration group), pear-shaped. 8. The gonad of 12-month-old fish (40 concentration group), shoulder pole-shaped. 9. The gonad of 12-month-old fish (25 concentration group), shoulder pole shaped, oogonia and spermatogonia appeared. 10. The gonad of 12-month-old fish (10 concentration group), cylindrical shaped. 11. The gonad of 12-month-old fish (60 concentration group), oocytes appeared. 12. The gonad of 15-month-old fish (40 concentration group), stage II ovary. (PGC-gonocytes, BV-blood vessels, OG-oogonia, SG-spermatogonia, PO-primary oocyte. The objective lens was 40 × in 1–7, 20 × in 8–11, 10 × in 12).

**Fig. 2:**
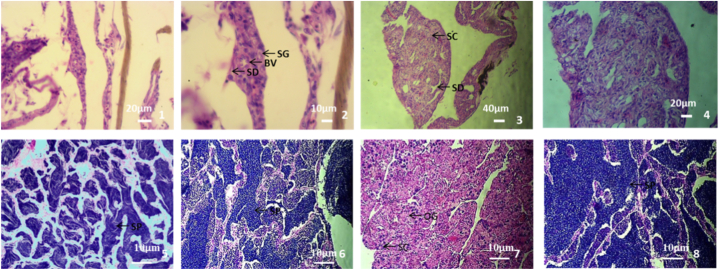
Gonad differentiation under testosterone propionate and high concentration 17α-MT in pond culture induction of *C. alburnus*. 1. The gonad of 5-month-old fish. 2. Figs. 2–1 amplified, found spermatogonia, spermatic duct and blood vessels. 3. The gonad of 12-month-old fish. 4. Figs. 2–3 amplified. 5. The gonad of 24-month-old fish. 6. The gonad of 36-month-old fish without ejaculation. 7. Another gonad of 36-month-old fish without ejaculation. 8. The gonad of 36-month-old fish capable of ejaculation (SG-spermatogonia, SD-spermatic duct, BV-blood vessels, SC-spermatocytes, SP-sperm. The objective lens was 40 × in 2, 20 × in 1 and 4, 10 × in 3 and 5–8).

**Fig. 3:**
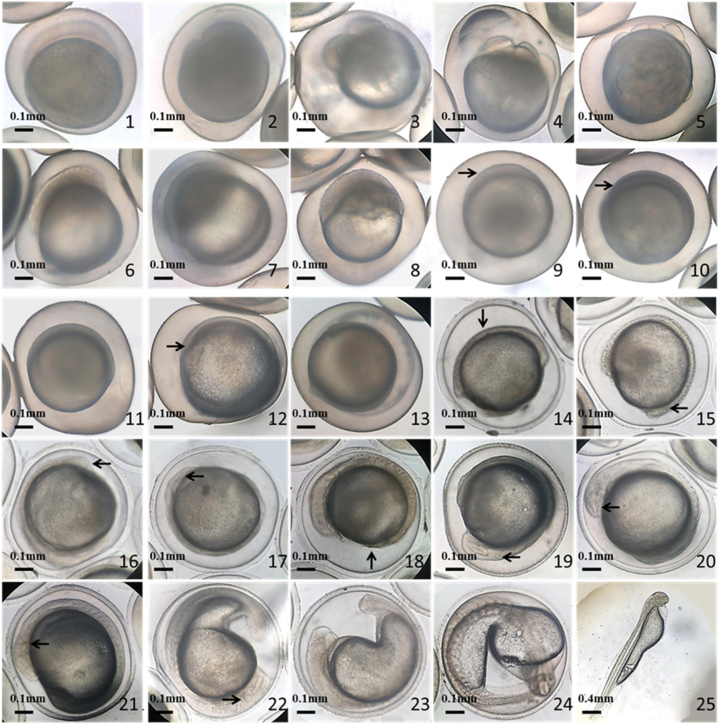
Embryonic development of AFC. 1. Blastoderm stage. 2. Two-cell stage 3. Four-cell stage. 4. Eight-cell stage. 5. Morula stage. 6. Early blastocyst. 7. Middle blastocyst. 8. Late blastocyst. 9. Early gastrulation, embryo ring (→). 10. Middle gastrulation, embryo shield (→). 11. Late gastrulation. 12. Neuro-embryonic stage, embryonic body rudiment (→). 13. Blastopore obturation. 14. Sarcomere stage, sarcomere (↓). 15. Ocular basal stage, eye base (oculogenin) (←). 16. Saccular stage, eye sac (←). 17. Olfactory plate stage, olfactory plate (←). 18. Tail-bud stage, tail bud (↑). 19. Auditory capsular stage, auditory capsule (←). 20. Crystal appearance stage, capsule crystal (←). 21. Cardiac stage, cardiac primordium (←). 22. Otolith stage, otolith (→). 23. Heartbeat stage. 24. Earlier exfoliation stage. 25. Newly hatched larvae.

**Fig. 4:**
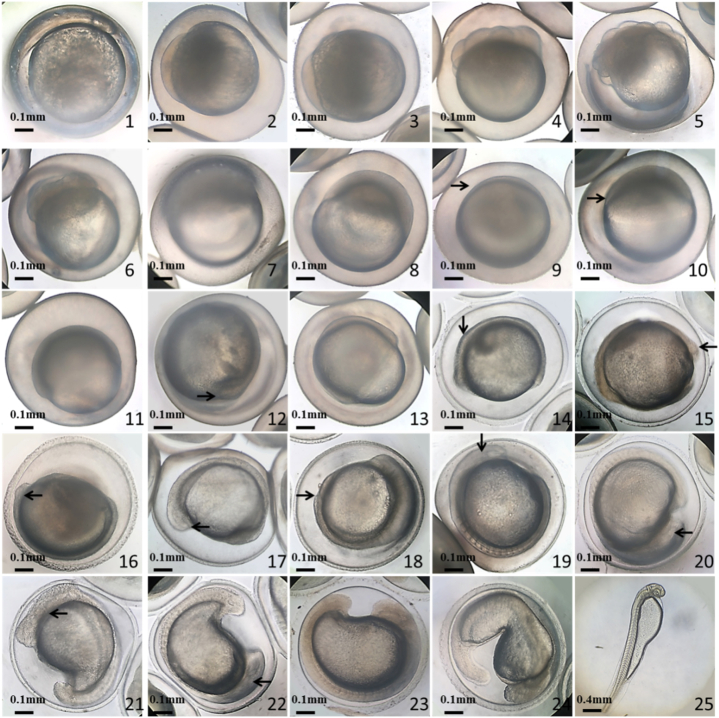
Embryonic development of common *C. alburnus*. 1. Blastoderm stage. 2. Two-cell stage 3. Four-cell stage. 4. Eight-cell stage. 5. Morula stage. 6. Early blastocyst. 7. Middle blastocyst. 8. Late blastocyst. 9. Early gastrulation, embryo ring (→). 10. Middle gastrulation, embryo shield (→). 11. Late gastrulation. 12. Neuro-embryonic stage, embryonic body rudiment (→). 13. Blastopore obturation. 14. Sarcomere stage, sarcomere (↓). 15. Ocular basal stage, eye base (oculogenin) (←). 16. Saccular stage, eye sac (←). 17. Olfactory plate stage, olfactory plate (←). 18. Tail-bud stage, tail bud (↑). 19. Auditory capsular stage, auditory capsule (←). 20. Crystal appearance stage, capsule crystal (←). 21. Cardiac stage, cardiac primordium (←). 22. Otolith stage, otolith (→). 23. Heartbeat stage. 24. Earlier exfoliation stage. 25. Newly hatched larvae.

**Fig. 5:**
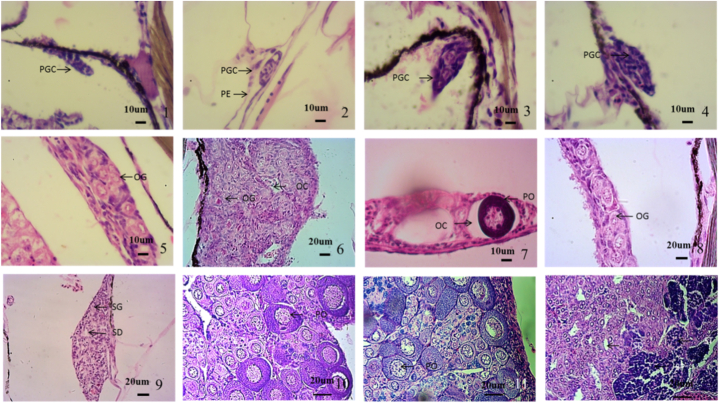
Gonadal differentiation. 1. The original gonad of 2-month-old all-female fish; 2. The original gonad of 2-month-old control fish; 3. The original gonad of 4-month-old all-female fish; 4. The original gonad of 4-month-old control fish; 5. The ovary of 6-month-old all-female 1# fish; 6. The ovary of 6-month-old all-female 2# fish; 7. The ovary of 6-month-old all-female 3# fish; 8. The ovary of 6-month-old control fish; 9. The testis of 6-month-old control fish; 10. The ovary of 13-month-old all-female fish; 11. The ovary of 13-month-old control fish; 12. The testis of 13-month-old control fish (PGC, gonocytes; PE, peritoneal epithelium; OG, oogonia; OC, ovary cavity; PO, primary oocyte; SD, seminiferous duct; SG, spermatogonial cell; ST, spermatozoa; SC, spermatocyte; SP, spermatozoa).

**Fig. 6:**
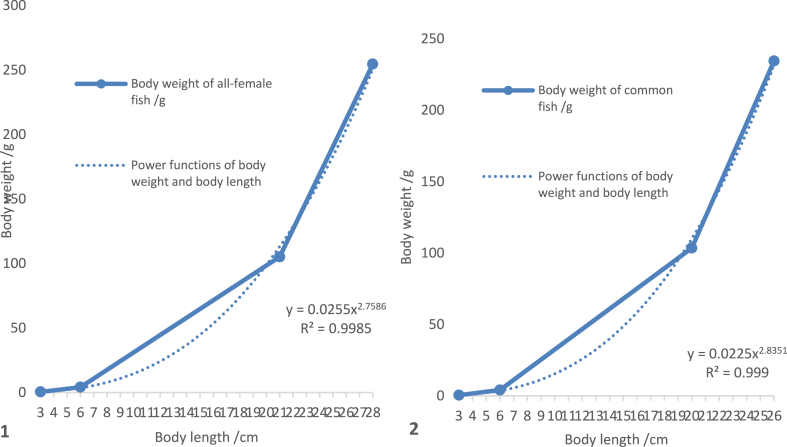
The correlation of body weight and length of 2- to 17-month-old *C. alburnus*. 1. The power functions of body weight and body length of all-female fish. 2. The power functions of body weight and body length of common fish.

**Fig. 7:**
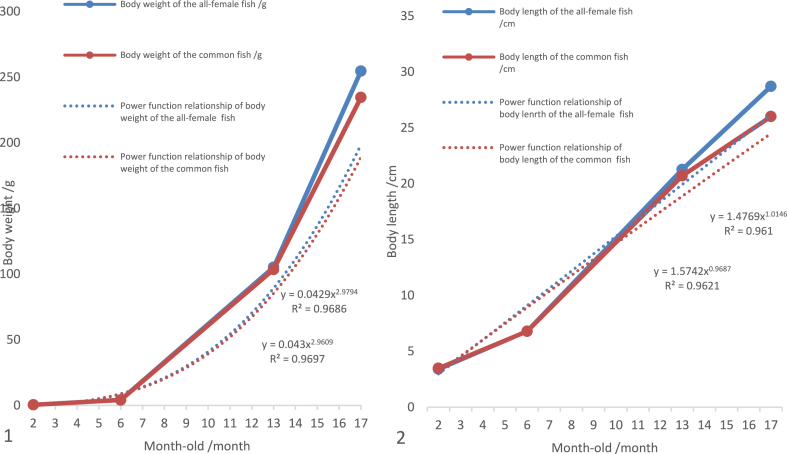
The correlation of body weight and length increasing with the age of the all-female and common *C. alburnus* at 2–17 months. 1. The power functions of body weight increasing with the age of the all-female and common fish. 2. The power functions of body length increasing with the age of the all-female and common fish.

The gonads of fish were observed at 2, 3, 5, 12, and 15 months, respectively. The results showed that before 5 months, there was no fully developed testis or ovary in the tissue section and males or females could not be distinguished. However, gonad differentiation could be analyzed from histologically. *C. alburnus* had two types of gonads at 2–3 months of age. Namely, a pear-shaped gonad and a spindle-shaped gonad. At 2 months, it could be observed that the pear-shaped gonad was relatively small, located on both sides of the body, and one end was suspended from the mesothelium on the peritoneal epithelium (Fig. 1). The spindle-shaped gonad was relatively large in size, long in shape, close to one side or the middle, and connected with the mesentery at both ends (Fig. 1–2). At 3 months, the morphological changes of these two types of gonads were relatively small. The pear-shaped gonads increased in size, became slightly elongated at both ends, and the gonocytes increased (Fig. 1, Fig. 2, Fig. 3). The spindle-shaped gonad increased in size. The gonocytes in the middle of the gonad were obvious (Fig. 1–4). By 5 months of age, gonad morphology had changed greatly and three types of gonads were present; namely, the pole-shaped gonad, the cylindrical gonad, and the pear-shaped gonad. The primordial pear-shaped gonad was rapidly stretched to both ends and was ready to develop into a testis. The gonad was shaped like a shoulder pole, similar to the spindle-shaped gonad, but longer and the width difference between the middle and both ends of the gonad was smaller than that of the spindle-shaped gonad. The primordial germ cell was located at the edge of the gonad, which differed from that of the spindle-shaped gonad which was located in the middle (Figs. 1–5). This type of typical gonad mainly appeared in the 40 (40-1) and 60 (60-1) concentration groups. The protospindle gonad was widened on both sides to form a cylindrical shape, which was thicker than the shoulder pole gonad. There were more gonocytes than those of the pear-shaped gonad, spindle-shaped gonad, and shoulder pole-shaped gonad. This gonad began to differentiate into the ovary (Fig. 1–6). This type of typical gonad mainly appeared in the 25 (25-1, 25-2) and 40 (40-2) concentration groups. Oogonia in the 40 concentration group (40-2) gonad developed into the ovary. Other concentration groups still produced pear-shaped gonads (Fig. 1–7). There were four types of gonads in the 10-, 25-, 40-, and 60-month-old fish, namely, the pole-shaped gonad, the pole-shaped intergonad, the cylindrical gonad, and the fully developed ovary. The pole-shaped gonad appeared in the 40-1 group, which was wider than the pole-shaped gonad of the 5-month-old fish, and the gonocytes was supposed to be increased (Figs. 1–8). However, there was still a tendency to develop into testis. The shoulder pole-shaped intersex gonad, which contained which both testis and ovarian characteristics, appeared in the 25-1 group (Figs. 1–9). The gonads of other experimental groups were cylindrical and fully developed ovaries. The area of the cylindrical gonad in the 12-month-old fish was supposed to be increasedlarger than that of the 5-month-old. Additionally, the number of gonocytes was supposed to be increased, blood vessels appeared, and the gonad developed into an ovary (Figs. 1–10). The fully developed ovaries appeared in the gonads of 40-2 and 60-2 groups. The fish of these two groups were supposed to be increased larger, and the gonads developed rapidly; oocytes appeared and fully developed ovaries formed (Figs. 1–11). At the age of 15 months only the fully developed ovaries were present and was filled with oocytes and oogonias (Figs. 1–12).

#### 3.1.2 Effect of testosterone propionate and high concentration 17α-MT in pond culture induction

The gonads of fish were observed at 5, 12, 24, and 36 months of age, respectively. The results showed that at 5 months, the gonads were all testis, and no ovary was found. The gonad was shoulder pole-shaped, which was wider in the middle than the pole-shaped gonad seen under low concentration 17α-MT treatment in indoor-net cage culture induction at the same age, producing spermatogonia, spermatic duct, and blood vessels (Figs. 2–1 and 2). The appearance of spermatogonia and spermatic duct indicated that the gonads had developed into testis at this time. For 12-month-old fish, the gonad was a fully developed testis, and spermatogonia or spermatocytes could be clearly observed (Figs. 2–3, Figs. 2–4). For the 24-month-old pond cultured fish, dissection confirmed that the fish were all male, one of which had obvious testis characteristics and more sperm (Figs. 2–5). For the 36-month-old pond cultured fish, it was found that five fish could extrude semen, but two were unable to. Anatomy of the gonads that one was a testis with normal development and sperm could be seen (Figs. 2–6), however, one which failed to extrude semen was found to be an intersexual gonad in which spermatogonia and oogonia could be observed at the same time (Figs. 2–7). The other five gonads which could extrude semen were found to be normal testes (Figs. 2–8). Three kinds of gonads were detected in the 36-month-old fish: ① normally developed testis which could extrude semen smoothly during the reproductive period; ② normal testis which could not extrude semen during the reproductive period; and ③ gonads with incomplete induction which degenerated to the ovary, forming an intersexual gonad.

### 3.2 Experiment II

#### 3.2.1 Embryonic development

Results showed that the embryonic development process could be divided into 27 stages according to external morphological characteristics (Table 1). At water temperatures of 24–26 °C, the first cleavage occurs 22 and 25 min after fertilization, sarcomeres appeared and organs formed 9 h 20 min and 9 h 35 min after fertilization. Hatching began approximately 25 h 25 min and 26 h 30 min after fertilization in the all-female group and common fish group, respectively. Hatching time in the common group seems to be shorter than the all-female group. Embryos in the all-female group developed normally (Figs. 3-1—3-25). Morphological characteristics did not differ from those of the common fish group (Figs. 4–1—4-25), however, the development of embryos in the all-female group was slightly slower than that of the common fish group (Table 1).

#### 3.2.2 Gonadal differentiation

Results showed that the gonadal differentiation of all-female fish was the same as that of common fish. A pair of primitive gonads were formed in the abdominal cavity of 2-month-old fish, which were located at the base of coelom below the kidney and above the intestine. There was one or a few primitive germ cells in the primitive gonad, which were oval in shape, larger in size, less basophilic in cytoplasm than the nucleus, less stained than the nucleus, and did not differentiate into spermatogonia or oogonia (Figs. 5–1 and 5–2). At the age of 4 months, the primordial gonad was enlarged, and the number of gonocytes was increased. With the proliferation of gonocytes, the primordial gonad also extended in the mesentery, but the gonocytes had not differentiated into spermatogonia or oogonia and the males and females could not yet be distinguished (Figs. 5–3 and 5–4). In the 6-month-old fish, the ovary could be distinguished from the testis. An ovarian cavity and oogonia could be seen in the ovary. According to the different development of the ovary, it could be found that there were individual oogonia, many oogonia, or primary oocytes in all-female fish ovaries. The volume of primary oocytes was obviously larger than that of the oocyte, which was oval, basophilic in cytoplasm, and dyed blue-purple by hematoxylin. Spermatogonia were present in the testis. The spermatogonia proliferated massively through mitosis, the number of cells increased significantly, and the structure of the spermatic duct appeared (Figs. 5–5—5-9).

It was found that most of the gonads of the all-female fish had completely differentiated into ovaries (23 all-female fish were randomly sampled, including 20 females and 3 undifferentiated; 8 common fish were randomly sampled, including 5 females and 3 males). At the age of 13 months, the characteristics of ovary and testis were obvious. Most ovaries were full of oocytes and oogonias, while testis were full of spermatogonia, spermatocytes, and spermatic duct. Some testis were full of sperm. It was also found that the gonads of all-female fish had developed into ovaries with obvious characteristics (48 all-female fish were randomly sampled, all females; 31 common fish were randomly sampled, including 21 females and 10 males) (Figs. 5–10—5-12).

#### 3.2.3 Growth analysis

① Comparison of growth indexes between all-female and common fish

Results showed that there was no significant difference in body weight and length between the all-female and common fish at 2 months of age (*P* > 0.05), however, the coefficient of variation of body weight and length of the all-female was higher than that of the common fish (Table 2). Under the same conditions, when the fish was raised to 6 months old, there was no significant difference between the all-female and common fish in body weight, body length, and other growth indicators (*P* > 0.05). Additionally, the coefficient of variation of body weight was also close to the common fish. However, the coefficient of variation of body length was opposite to that at 2 months, being lower than that of the common fish (Table 2). At the age of 13 months, there was no significant difference in body weight and length between the all-female and common fish (*P* > 0.05), but the coefficients of variation of body weight and length were lower than those of the common fish (Table 2). At the age of 17 months, the body weight and length of all-female fish were significantly higher than that of common fish (*P* < 0.05) (Table 2). The coefficient of variation of body weight and length were significantly lower than those of common fish (*P* < 0.05).

② Relationship between body weight and body length

A curve regression was conducted between the measured average body length and body weight of the all-female (Figs. 6–1) and common fish (Figs. 6–2) at different ages. The results showed that the power functions of body weight and body length of all-female and common fish were W = 0.0255 L^2.7586^ (R^2^ = 0.9985) and W = 0.0225 L^2.8351^ (R^2^ = 0.9990) (Fig. 6).

③ Relationship between body weight, body length, and month age

Our results showed that the power function relationship of body weight increasing with the age of the all-female and common fish at 2–17 months was as follows: W = 0.0429t^2.9794^ (R^2^ = 0.9686) and W = 0.043t^2.9609^ (R^2^ = 0.9697) (Figs. 7–1). The power function relationship of body length increasing with the age of the all-female and common fish at 2–17 months was as follows: L = 1.4769t^1.0146^ (R^2^ = 0.9610) and L = 1.5742t^0.9687^ (R^2^ = 0.9621) (Figs. 7–2).

## 4 Discussion

### 4.1 Low concentration 17α-MT in indoor-net cage culture induction

In this study, the pear-shaped gonad in 2-month-old fish differentiated into testes, and the spindle-shaped gonad in differentiated into ovaries, similar to that of *V. vimba*, *C. auratus*, and *M. anguillicaudatus* [4,12,14]. In 2- to 3-month-old fish, only pear-shaped gonads were observed in the 60 concentration group, while two types of gonads were found in other concentration groups, indicating that before 3 months, a higher concentration (60 μg/g) of 17α-MT had the most significant effect on gonadal differentiation. Previous studies have shown that, suitable concentration of 17 α- MT had the most significant effect on gonadal histology of fish [4,9]. The results of gonadal differentiation in 5-month-old fish showed that, except for some pear-shaped gonads that did not further differentiate, other pear-shaped gonads were differentiated into testis under 40 days of a higher concentration (40 and 60 μg/g) of 17 α-MT feeding + 70 days of normal feeding. The differentiation and development of the ovary of most teleost fishes were earlier than that of testis [9,10]. Spindle-shaped gonads had turned into cylindrical gonads at the age of 5 months, which indicated that under 40 days of 25 μg/g 17 α-MT feeding +70 days of normal feeding, 40 days of 40 μg/g 17 α-MT feeding +70 days of normal feeding, or 60 days of hormone feeding +50 days of normal feeding, the gonad had not been induced by androgen to transform into testis, or the transformation was incomplete and the gonad degenerates into an ovary. However, the gonads of the 10 concentration group were all pear-shaped, which might be related to the smaller size of the fish in the 10 concentration group, slow development, and the later development of the testis than the ovary. At 12 months, the shoulder pole-shaped gonad still maintained the trend of differentiation into testis, indicating that treatment with 40 days of 40 μg/g 17 α-MT feeding +70 days of normal feeding would result the closest to successful sex reversal, which was similar to the results of Liu et al. [4]. Namely, 50 ng/L 17 α- MT had the most significant effect on gonadal histology of fish, however, whether this medium concentration could be reversed successfully still needs follow-up experimental verification. The shoulder pole-shaped intersex gonad appeared in the 25-1 group, and the reason for this might be that the gonad with the tendency to differentiate into ovary in the early stage appeared in the 25-1 group after treatment of 17α-MT and its reversal to the testis was incomplete. Subsequently, the gonad degenerated into the ovary, forming a gonad with both testis and ovarian characteristics. This phenomenon had also been found in previous research, and they speculated that the reason for this phenomenon might be due to hormone treatment causing a brief change in gonadal differentiation status, or it might be due to inappropriate dosage or duration of hormone treatment [24].

Chen et al. [12] found that under high temperature conditions, *Misgurnus anguillicaudatus* was hermaphroditic, that is, some individuals' gonads were flat, and there were traces of ovarian cavity in the gonads, but only spermatogonia and spermatocytes at all levels were present in the gonads. However, in the gonads of other individuals, there were oogonia, early primary oocytes, spermatogonia, and other types of spermatocytes, thus producing these different types of flat interzonal gonads under environmental stress. The intersex gonad in this experiment was with both oogonia and spermatogonia. Until the age of 15 months, they were fully developed into ovaries. Therefore, we believed that the differentiation trend resulting from a low concentration of 17α-MT may be because under the stimulation of 17α-MT, some gonads had a tendency to incompletely transform into testis. There were three types of gonads in 5-month-old fishes and four types of gonads in 12-month-old fishes, however, they all differentiated into ovaries in 15-month-old fishes. This shows that the concentration or method of hormone treatment in the indoor still needed to be optimized.

### 4.2 Testosterone propionate and high concentration 17α-MT in pond culture induction

At the age of 5 months, the gonads of *C. alburnus* cultured in pond had developed into testes, which indicated that after 5 months of age, the gonads would differentiate into testes through androgen induction and pond culture. Compared with the three types of gonads of 5-month-old indoor cage-cultured fish, we speculated that there were two reasons for the better androgen induction effect of pond cultured fish. First, the treatment methods were different. The treatment method of the outer pond was from a low to high concentration, with the maximum concentration set at 140 μg/g, far higher than the maximum concentration 60 μg/g induced in the indoor. Second, the culture environment of the outer pond was more superior, and the fish would grow rapidly. At the age of 5 months, the fish would have grown to a certain extent (body weight 2.53 ± 0.12 g, body length 83.13 ± 5.2 mm). Comparing the growth of indoor cage-cultured fish (body weight 1.12 ± 0.39 g, body length 46.66 ± 3.50 mm) with the differentiation and development of gonads, the rate of gonad differentiation and development was consistent with the size of the body, and the indexes such as body weight and body length had a certain correlation with the degree of gonad differentiation and development. Under the condition that the environment was more favorable for growth, gonad differentiation and development of *C. alburnus* was earlier, and the degree of gonad differentiation and development might have a certain impact on the induction effect., which had also been elaborated in previous research [1,3,9]. Li et al. [8] believed that after MT treatment, the gonads of *Epinephelus akanta* went through four stages: 1) in the early stage of sex reversal, spermatogonia proliferated; 2) at the middle stage of sexual reversal, spermatogonia and degenerated phase II oocytes co-existed, but the testis tissue played a dominant role in the gonad; 3) with the development of sex reversal, spermatogonia and spermatocytes became the dominant cells in the gonad; and 4) the testis was full of sperm. It was also found that the histological structure of the testis of male sex reversal fish was similar to that of normal male fish. Some fish turned into functional male fish and could ejaculate after slight abdominal compression. However, after a high dose or long-term 17α-MT treatment, the induced sex reversal male semen might not have the ability to inseminate. In this study, the gonad of *C. alburnus* that was successfully induced by androgen treatment might also have gone through the four stages described by Li et al. [8], and finally the testis was filled with sperm. Additionally, it was also found that the functional male fish that could extrude semen and the sex reversal male fish that could not extrude semen, and the latter could not extrude semen, which might be related to the abnormal gonadal function or delayed development caused by high-dose or high-intensity hormone treatment. Olito and Brock [24] treated *Salmo gairdneri* with MT, and some individuals that changed into males would undergo a reversal, becoming facultative fish or female fish. They speculated that the reason for this phenomenon might be that hormone treatment caused a temporary change in gonadal sex differentiation, or it might be caused by inappropriate dosage or duration of hormone treatment. Facultative fish indicated that the role of hormones in changing the sex of fish is not permanent in all individuals. Therefore, the results of this test showed that there might be four situations in the breeding process of *C. albertus* resulting from pseudo-male induction at the initial stage: ① a functional pseudo-male with normal testis development which could successfully extrude semen during the breeding period; ② a pseudo-male with normal testis development, but unable to extrude semen during the breeding period; ③ incomplete induction wherein the gonad degenerated to the ovary, resulting in a facultative fish with an intersexual gonad; and ④ incomplete induction wherein the gonad degenerated into an ovary and resulted in a female fish (this kind of gonad was not found in the pond, but was found in the indoor cage). Treatment with testosterone propionate and high concentration 17α-MT in pond culture induction could avoid complete degeneration of the gonads into ovaries, and the effect was better. However, a few gonads became intergonads, indicating that this method still needed to be further optimized to improve the success rate of induction.

### 4.3 Reproduction and development of all-female fish

The embryo development of all-female fish was normal and the time spent in each phase was similar to that of the common fish group. Additionally, the morphological characteristics of embryos in each time sequence were similar to that of the common fish group, except that the embryo development rate was slightly slower than that of the common fish group. This showed that a series of measures to cultivate the all-female *C. alburnus* did not prevent the embryo from developing at its normal rate. Jiang et al. [25] also showed that the larvae of *P. olivaceus* hatched later from embryos would become female fish than from ordinary embryos, and the growth rate of seedlings would become female fish was higher than that of ordinary seedlings. Whether this indicated that there was some relationship between the hatching rate of embryos would become all-female fish and the growth rate of hatching fry needed further research to prove.

The gonadal development of fish was mainly characterized by anatomy and cytology [26]. At the anatomical level, there were mainly ovarian cavity and spermatic duct. At the cytological level, it mainly included the appearance of oocytes and spermatogonia, oocytes, and spermatocytes [27]. In this study, the characteristics of oogonia (spermatogonia), oocytes (spermatogonia), ovarian cavity, and spermatic duct were used as markers to distinguish the among types of gonads. The gonads of the all-female and common fish did not show these marker cells or structures before the age of 4 months, indicating that the testis or ovary were not differentiated. At the age of 6 months, the gonads had been differentiated, and there were only individual oocytes, more oocytes, and three stages of development of ovaries with primary oocytes. Therefore, it was speculated that the main differentiation time of gonads of all-female and common fish was between 4 and 6 months old. At the same time, this study compared the ovarian development of the all-female fish with that of the common fish and found no difference in the development characteristics between them; the gonads of the all-female fish had all developed into ovaries with obvious characteristics at the age of 13 months. This showed that a series of measures to cultivate all-female fish could ensure the full female sex without affecting the normal development of gonads.

### 4.4 Growth characteristics of all-female fish

Body weight, body length, and coefficient of variation were important indicators of fish growth [28]. Comparing the changes of these indicators between the all-female and common fish at the age of 2–17 months, it was found that with increasing age, there was no significant difference in body weight and body length between the all-female and common fish. From the age of 2–13 months (*P* > 0.05) to the age of 17 months, the body weight and body length of the all-female were significantly greater than the common fish (*P* < 0.05), indicating that the all-female *C. albertus* grew faster than the common fish. This was consistent with the growth test results of *Ctenopharyngodon idella* [29] and *C. auratus* [30]. Additionally, in the process of fish growth, there was a certain correlation between body length and body weight, and between body weight and body length and age [22,23]. In the power function relationship, the index b value could not only judge whether fish were growing at an allometric or isokinetic rate, but also reflected the breeding environment and nutritional conditions of fish [31,32]. If the index b value was close to 3, the population was growing at a uniform rate [33]. The results of this study showed that the b value in the power function relationship of the body weight and body length of all-female and common fish was close to 3, indicating that the growth rate of all-female and common fish was similar to that under pond culture conditions. It was speculated that the pond culture environment was suitable for the growth of *C. albertus*. The R^2^ in the power function of body weight and body length of all-female and common fish increased with age and was close to 1, indicating that there was a strong correlation between body weight, body length, and age. Therefore, based on the data of body weight and body length, relationship between body mass and body length, and relationship between body mass, body length, and age of all-female and common fish, we believed that the correlation between body weight, body length and month age was strong and the all-female fish had a faster growth rate than the common fish when the all-female and common fish grew under suitable conditions. Modern aquaculture requires rapid growth of aquaculture varieties and consistency of growth specifications [34]. The coefficient of variation of body weight and body length was one of the main indicators reflecting the consistency of fish growth specifications, which indicated the degree of genetic variation of the population in terms of genetics [35]. The results showed that the coefficient of variation of body weight and body length of all-female fish was higher than that of common fish at 2 months. The coefficient of variation of body weight at 6 months was close to that of common fish, but the coefficient of variation of body length was opposite to that of 2 months old, which was lower than that of common fish. At 13–17 months, this value was lower than that of common fish. This shows that with the growth of all-female fish, the consistency of specifications was better than that of common fish. The smaller the difference in commodity specifications of adult fish, the higher the breeding efficiency would be [34,35], so the breeding of new varieties of all-female fish would meet the requirements of modern aquaculture. At the same time, because the growth consistency could show the homogeneity of genes among populations to a certain extent, the higher the homozygosity of genes, the smaller the genetic variation, and the better the growth consistency [34,35], it was speculated that the low coefficient of variation of all-female fish might be related to its high genetic purity.

## 5 Conclusion

In this study, we observed that a low concentration 17α-MT in indoor-net cage culture induction was not effective. However, testosterone propionate and high concentration 17α-MT in pond culture induction had a good effect, resulting in a functional pseudo-male with normal testis development which could successfully extrude semen during the breeding period. Additionally, compared with common *C. alburnus*, all-female embryo had normal embryonic development, the development time of each stage and morphological characteristics were basically the same, and the gonadal differentiation of all-female fish were normal. The correlation between weight, length, and age of all-female and common fish was strong. All-female fish had a faster growth rate and more uniform growth specification than common fish.

## Funding

This research was supported by the Cultivation and demonstration of new varieties of *culter alburnus* with high quality and stress resistance (2021C02069-3).

## Compliance with ethical standards

This study was approved by the Ethics Committee of Laboratory Animal Center of Zhejiang Institute of Freshwater Fisheries. The animal protocols were approved by the Institutional Animal Care and Use Committee (IACUC) of the Zhejiang Institute of Freshwater Fisheries. The animal study was reviewed and approved according to the guidelines of the Animal Experiment Committee, Zhejiang Institute of Freshwater Fishery (ZIFF20200301).

## Data availability statement

Data will be made available on request.

## Compliance with ethical standards

This study was approved by the Ethics Committee of Laboratory Animal Center of Zhejiang Institute of Freshwater Fisheries.

## Code availability

Software application.

## CRediT authorship contribution statement

**Shun Cheng:** Writing – review & editing, Writing – original draft, Resources, Project administration, Methodology, Investigation, Funding acquisition, Formal analysis, Data curation, Conceptualization. **Mei-li Chi:** Methodology, Investigation, Formal analysis, Data curation. **Shi-li Liu:** Validation, Methodology, Investigation. **Jian-bo Zheng:** Visualization, Validation, Formal analysis. **Wen-ping Jiang:** Validation, Resources. **Xiao-ying Hang:** Visualization, Methodology. **Miao Peng:** Visualization, Data curation. **Fei Li:** Supervision, Project administration, Conceptualization.

## Declaration of competing interest

The authors declare the following financial interests/personal relationships which may be considered as potential competing interests:The authors reports was provided by Zhejiang Institute of Freshwater Fisheries. The authors reports a relationship with Zhejiang Institute of Freshwater Fisheries that includes: board membership. Li Fei has patent pending to Licensee. The authors declare that there are no competing interests. If there are other authors, they declare that they have no known competing financial interests or personal relationships that could have appeared to influence the work reported in this paper.
